# Myeloid-Derived Suppressor Cells in Sepsis

**DOI:** 10.3389/fimmu.2019.00327

**Published:** 2019-02-27

**Authors:** Irene T. Schrijver, Charlotte Théroude, Thierry Roger

**Affiliations:** Infectious Diseases Service, Department of Medicine, Lausanne University Hospital, Epalinges, Switzerland

**Keywords:** sepsis, infectious disease, innate immunity, myeloid-derived suppressor cells, biomarker, immunosuppression, inflammation, personalized medicine

## Abstract

Myeloid-derived suppressor cells (MDSCs) are immature myeloid cells characterized by their immunosuppressive functions. MDSCs expand during chronic and acute inflammatory conditions, the best described being cancer. Recent studies uncovered an important role of MDSCs in the pathogenesis of infectious diseases along with sepsis. Here we discuss the mechanisms underlying the expansion and immunosuppressive functions of MDSCs, and the results of preclinical and clinical studies linking MDSCs to sepsis pathogenesis. Strikingly, all clinical studies to date suggest that high proportions of blood MDSCs are associated with clinical worsening, the incidence of nosocomial infections and/or mortality. Hence, MDSCs are attractive biomarkers and therapeutic targets for sepsis, especially because these cells are barely detectable in healthy subjects. Blocking MDSC-mediated immunosuppression and trafficking or depleting MDSCs might all improve sepsis outcome. While some key aspects of MDSCs biology need in depth investigations, exploring these avenues may participate to pave the way toward the implementation of personalized medicine and precision immunotherapy for patients suffering from sepsis.

## Introduction

Sepsis is one of the leading causes of preventable death. Sepsis is defined as a “life-threatening organ dysfunction caused by a dysregulated host response to infection” (1). The mortality rate of sepsis accounts for five-to-six million deaths of ~30 million cases per year worldwide. Sepsis incidence is rising due to the aging of the population, the burden of chronic diseases, the increasing number of immunocompromised patients, and the resistance of microorganisms to antimicrobials (2). In 2017, the World Health Assembly and the World Health Organization made sepsis a global health priority by adopting a resolution to improve the prevention, diagnosis, and management of sepsis.

Innate immune cells, such as monocytes/macrophages, dendritic cells (DCs), and neutrophils, sense microbial and danger-associated molecular patterns (MAMPs produced by microorganisms, and DAMPs released by injured or stressed cells) through pattern recognition receptors (PRRs). PRRs are grouped into five main families: toll-like receptors (TLRs), NOD-like receptors, C-type lectins, scavenger receptors, RIG-I-like receptors, and intra-cytosolic DNA sensors (3). The interaction between PRRs and MAMPs or DAMPs triggers intracellular signaling pathways that coordinate gene expression, the development of the inflammatory response, the establishment of antimicrobial cellular and humoral responses, and the restoration of homeostasis once pathogens have been contained or eradicated. Sepsis is characterized by an early exacerbation of antimicrobial defense mechanisms, the so-called hyper-inflammatory “cytokine storm,” mediating tissue injury, organ dysfunctions and early mortality, and a concomitant shift toward inflammation resolution and tissue repair. Sepsis-induced immunoparalysis (or immunosuppression) favors the development of secondary infections and long-term immune disabilities accounting for late mortality (4–8).

During the last decades, early goal-directed therapy decreased early mortality from sepsis, which contributed to shift the sepsis ICU population toward a population suffering from chronic critical illness (CCI). Indeed, a subset of ICU patients surviving sepsis develop CCI characterized by long-lasting immunosuppression associated with a persistent, low-grade, inflammation maintained by the continuing release of DAMPs. The underlying inflammation is associated with catabolism and malnutrition. The term persistent inflammation-immunosuppression and catabolism syndrome (PICS) has been proposed to characterize this degraded state. PICS is associated with long-term morbidity, late multiple organ failures and late mortality (9–11).

Clinical trials testing adjunctive therapy to dampen inflammation-related dysfunctions in sepsis have not been conclusive (12). Several reasons may account for these failures, among them the large heterogeneity of the sepsis syndrome. Nowadays, the prevalent view is that restoration of immune capacities using immuno-stimulants might be more efficient than anti-inflammatory therapies. In any case, personalized medicine should be used to define at an individual level whether inflammatory cytokines, immunoparalysis, or metabolism has to be targeted (4, 7, 13–17). In that perspective, significant efforts are devoted to the identification of genetic, molecular, and cellular biomarkers to stratify patients for clinical studies and treatment based on clinical condition and disease stage.

We poorly understand what is responsible for a dysregulated host response and the delay returning to homeostasis in sepsis patients (4–8, 18). Growing interest focuses on a subpopulation of leukocytes called myeloid-derived suppressor cells (MDSCs). MDSCs are involved in the regulation of the immune response in many pathological situations, the best-studied being cancer. A number of comprehensive reviews discusses MDSCs in the context of cancer, autoimmunity and infectious diseases [see for example (19–26)]. Interestingly, recent data suggest that MDSCs are involved in immune dysfunctions observed in sepsis. In this review, we summarize and discuss our current knowledge about the role played by MDSCs during sepsis and the potential of using MDSCs as biomarkers and therapeutic targets of sepsis.

## Myeloid-Derived Suppressor Cells (MDSCs)

MDSCs are immature myeloid cells that expand during chronic and acute inflammatory conditions. The premises of MDSC discovery date back more than a century when tumor progression was associated with extra-medullary haematopoiesis and neutrophilia. In the mid-1960s, Lappat and Cawein reported that subcutaneously transplanted A-280 tumor cells generate factors involved in a leucocytosis response that sustains tumor growth (27). Subsequently, leucocytosis was involved in the expansion of cells of myeloid origin with immunosuppressive activity (24). These cells express reduced levels of conventional markers for mature myeloid and lymphoid cells and were named natural suppressor cells, null cells, immature myeloid cells, or myeloid suppressor cells. In 2007, “myeloid-derived suppressor cells” was adopted as a unifying term to minimize the confusion prevailing in the literature (28).

MDSCs are defined primarily by their immunosuppressive functions. Within sepsis, one may predict that MDSCs play a dual role depending on disease progression. On the one hand, MDSCs may be beneficial by limiting hyper-inflammation during the early stages of sepsis, hence protecting from early organ dysfunction. On the other hand, MDSCs may be detrimental by amplifying long-term immunosuppression and contributing to CCI and/or PICS (8, 10). As discussed later, these two facets have been highlighted in experimental models, while clinical studies all pointed to a deleterious role of MDSCs.

Minimal phenotypic characteristics of MDSCs have been proposed, but a definite, consensual phenotyping scheme is lacking (29, 30). Two main subpopulations of MDSCs are usually considered: polymorphonuclear MDSCs (PMN-MDSCs, previously called granulocytic-MDSCs) and monocytic MDSCs (M-MDSCs), so-called because of their morphological and phenotypical homologies with PMNs and monocytes (26, 29–32). In mice, MSDCs are defined as Gr1^+^ CD11b^+^ cells (Gr1: granulocyte receptor-1 antigen, consisting of Ly-6G and Ly-6C antigens). PMN-MDSCs are CD11b^+^ Ly6G^+^ Ly6C^low^ cells and M-MDSCs CD11b^+^ Ly6G^−^ Ly6C^high^ cells. In humans, PMN-MDSCs are CD11b^+^ CD14^−^ CD33^+^ (CD15^+^ or CD66^+^) cells and M-MDSCs CD11b^+^ CD14^+^ HLA-DR^low/−^ CD15^−^ cells. PMN-MDSCs overlap phenotypically with mature neutrophils but contrary to PMNs, MDSCs sediment within the PBMC fraction in ficoll gradients after density separation of whole blood. Whether low density gradient (LDGs) PMNs and PMN-MDSCs are the same entity is unclear, albeit the terms is used interchangeably in the literature. The identification of PMN-MDSCs by density gradient is further limited by the rise of not only low-density neutrophils, but also high-density CD62L^dim^ neutrophils that suppress T cells in the blood of healthy humans infused with endotoxin (33). Additional markers are proposed to differentiate MDSCs from monocytes or granulocytes, for example high expression of lectin-type oxidized LDL receptor-1 (LOX-1) by PMN-MDSCs when compared to granulocytes in whole blood (33, 34).

Complicating the picture, other MDSC subsets have been described, among others early-stage MDSCs (e-MDSCs) and eosinophilic MDSCs (eo-MDSCs) (29, 35). In addition, tumor-associated macrophages (TAMs), which unlike their name suggests are present in inflammatory conditions bedsides cancer, can be considered as one of the members making up the MDSC spectrum (36, 37). Finally, MDSCs are highly plastic. They can differentiate into osteoclasts and non-suppressive mature myeloid cells, and M-MDSCs can differentiate into TAMs and PMN-MDSCs (38–41). Overall, to this day, identifying MDSCs based on cell surface phenotyping usually ends up with a mixed population, eventually containing other myeloid cell types, that does not take into account the hallmark immunosuppressive function of MDSCs.

Adding to the above caveats, improper cell separation through density gradient and freezing whole blood or PBMC samples before flow cytometry analyses affects the detection of MDSCs, especially PMN-MDSCs. Hence, an objective of future studies is to optimize and harmonize sample handling and flow cytometry strategies (labeling, gating, and analyses) to quantify MDSCs in whole blood. This will facilitate the comparison of results from different studies to determine whether MDSCs are reliable disease biomarkers (32, 42). Strategies to identify cell surface markers discriminating MDSCs from other leukocytes using unbiased high discriminating techniques like RNA sequencing and mass cytometry analyses are starting to be used and have not yet improved the immuno-phenotyping of MDSCs (43). To summarize, the analysis of MDSCs and comparing results from different studies is complicated mainly because of: (1) the functional definition of MDSCs, (2) the lack of a defined phenotype(s) of MDSCs, and (3) the plasticity of MDSCs.

## MDSCs Expansion and Activation

**Figure 1:**
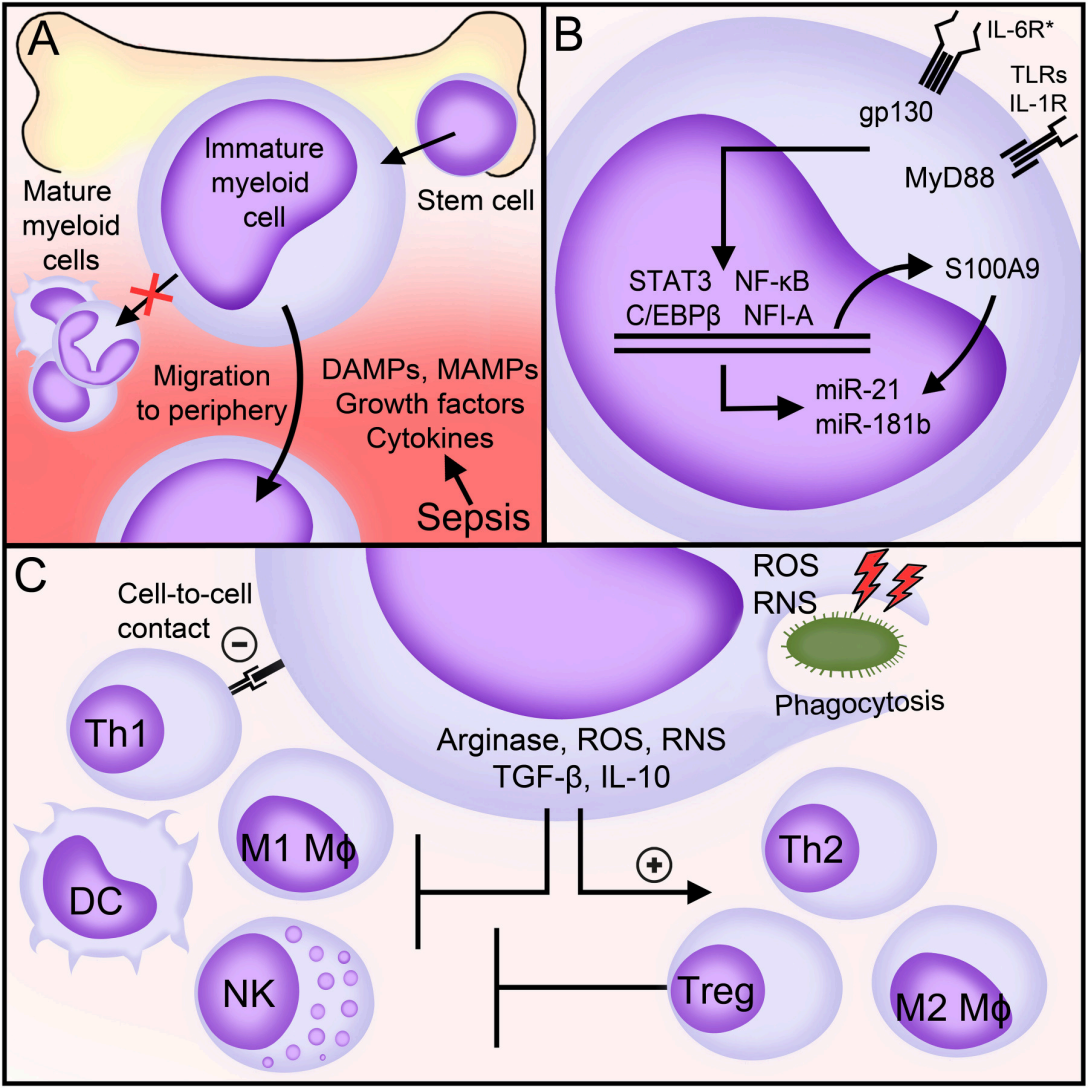
MDSCs in sepsis. **(A)** Factors generated during sepsis induce the expansion and egress of MDSCs from the bone marrow into the peripheral blood. **(B)** Main signaling pathways involved in the expansion and the immunosuppressive functions of MDSCs during sepsis. **(C)** Biological functions of MDSCs during sepsis. See body text for detailed explanations. DAMPs, danger-associated molecular patterns; MAMPs, microbial-associated molecular patterns; IL-6R^*^, interleukin (IL)-6 receptor family of cytokines (commonly referred to as gp130 cytokines); gp130, glycoprotein 130; TLRs, toll-like receptors; IL-1R, interleukin-1 receptor; MyD88, Myeloid differentiation primary response 88; NF-κB, nuclear factor-κB; NFI-A, nuclear factor I A; STAT, signal transducer and activator of transcription; miR, microRNA; Mφ, macrophage; DC, dendritic cell; Th, T helper; NK, natural killer; Treg, T regulatory; IFNγ, interferon γ; ROS, reactive oxygen species; RNS, reactive nitrogen species; TGF-β, transforming growth factor-β; IL-10, interleukin-10.

Hematopoietic stem cells differentiate into common myeloid progenitors giving rise to immature myeloid cells. An inflammatory environment, as observed in sepsis, stimulates the egress of immature myeloid cells from the bone marrow into the blood stream and the gain immunosuppressive functions (26, 44) (Figure 1). The identification of mediators and molecular mechanisms underlying the expansion and the immunosuppressive functions of MDSCs may pinpoint to original therapeutic targets for various diseases. Most of our knowledge comes from disease conditions other than sepsis. In sepsis, most relevant studies analyse the impact of gene specific knockout or the infusion of MDSCs in mice exposed to polymicrobial sepsis induced by cecal ligation and puncture (CLP).

In mice subjected to CLP, MDSCs accumulate in secondary lymphoid organs, in which they represent as much as 10–20% of all leukocytes (45). In the spleen, MDSCs expand within 3–5 days, culminate after 10–14 days and stay high for at least 12 weeks. The rise of MDSCs appears to be a complex and progressive process that involves expansion and activation of immature myeloid cells through many factors. These factors are not specific to sepsis and can be redundant. The expansion of immature myeloid cells is primarily mediated by the action of growth factors (GF) and colony stimulating factors (CSF) [such as vascular endothelial-GF (VEGF), granulocyte-macrophage-CSF, macrophage-CSF (M-CSF) and stem cell factor (SCF)], DAMPs (S100 calcium-binding protein A8/A9, S100A8/9), and possibly chemokines (CXCL1, CXCL2). Activation of pathogenic MDSCs is induced by MAMPs (LPS, staphylococcal enterotoxins), DAMPs (HMGB1), cytokines (IFNγ, IL-1β, IL-4, IL-6, IL-7, IL-10, IL-13, TNF, CXCL3), and acute phase proteins (α2-macroglobulin, serum amyloid A) (26, 42, 46–56). These same factors may induce the maturation of MDSCs, with possible different outcomes. For example, M-MDSCs exposed to R848 (a TLR7/8 agonist), TNF and IFNγ differentiate into inflammatory macrophages that produce TNF and IL-12, while M-MDSCs exposed to Pam_3_CSK_4_ (a TLR1/2 agonist) differentiate into immunosuppressive macrophages producing IL-10 (47, 57).

Myeloid differentiation primary response 88 (MyD88), glycoprotein 130 (gp130) and nuclear factor I A (NFIA, a transcription factor) control the expansion and the immunosuppressive functions of MDSCs (Figure 1). MyD88 is an adaptor molecule that initiates quick nuclear factor-κB (NF-κB) signaling through the IL-1 receptor and all TLRs except TLR3. gp130 is a signal transducer co-receptor for IL-6 family cytokines that cooperates with signal transducer and activator of transcription (STAT3) and C/EBPβ to upregulate MDSCs (45, 54). MDSCs do not expand in MyD88^−/−^ germline mice and in hepatocyte-specific gp130^−/−^ and myeloid-specific Nfia^−/−^ mice subjected to CLP (25, 45, 49, 58, 59). Additionally, Gr1^+^ CD11b^+^ MDSCs lacking NFI-A lose their immunosuppressive functions and stop differentiating into mature myeloid cells. The expansion of MDSCs is normal in myeloid-specific Cebpb^−/−^ septic mice, but Cebpb^−/−^ MDSCs produce reduced levels of IL-10 (52, 60). During CLP, triggering of a NF-κB/C/EBPβ/STAT3 axis upregulates the expression of S100A9 (also known as calgranulin B). S100A9 translocates into the nucleus to upregulate the transcription of microRNAs miR-21 and miR-181b that fine tune the expansion and the functions of MDSCs. Mice lacking S100A9 have less splenic and bone marrow MDSCs especially during late sepsis and are protected from death (61, 62). *In vivo* blockade of miR-21 and miR-181 decreases bone marrow MDSCs and improves sepsis survival (63). Recent work suggest that Nfe2l2 (nuclear factor, erythroid derived 2, Like 2; also known as NRF2) contributes to increase the metabolic activity and the expansion of Gr1^+^ CD11b^+^ MDSCs during endotoxemia (64).

The molecules mentioned above are not specific to MDSCs, and their genetic ablation can influence other arms of the defenses systems. To bypass this limitation, MDSCs isolated from sepsis mice are infused into wild-type recipient mice subjected to microbial insults. The adoptive transfer of Gr-1^+^ CD11b^+^ MDSCs or PMN-MDSCs harvested from septic donor-mice into recipient mice protects the later from acute endotoxemia, rapidly lethal CLP and *Pseudomonas* airway infection (54, 60, 65–68). Two studies compare the benefits provided by the infusion of Gr-1^+^ CD11b^+^ MDSCs taken either quickly or late after the onset of infection (i.e., 3 vs. 10–12 days post-infection). Interestingly, the transfer of early MDSCs increases while the transfer of late MDSCs decreases or does not change mortality (65, 69). Supported by additional *in vivo* and *in vitro* data (65, 69), this can be explained by the fact that, during the course of sepsis, MDSCs evolve to a more immature and anti-inflammatory state. More work will be required to appraise how much the maturation stage of MDSCs, the timing of expansion and/or infusion of MDSCs and the severity of the infectious models tip the balance toward a beneficial or a detrimental impact of MDSCs on sepsis outcome. As we will see in the last paragraph, the picture is clearer in clinical settings where high proportions of MDSCs indicate a poor prognosis.

The main epigenetic mechanisms, i.e., DNA methylation, histones methylation and acetylation, miRNAs and long non-coding RNAs (LncRNAs), have been implicated in the development of MDSCs with different outcomes (70). For example, inhibition of the DNA methyltransferases (DNMTs) 3a and 3b promotes the suppressive functions of MDSCs while inhibition of the histone methyltransferase SETD1B limits their suppressive function (71, 72). Pan-inhibitors of histone deacetylases (HDACs) 1–11 elicit robust expansion of M-MDSCs (73), in agreement with the observation that HDAC11 itself acts as a negative regulator of expansion and function of MDSCs (74). Interestingly, HDAC2 drives the phenotypic differentiation of M-MDSCs into PMN-MDSCs in tumor bearing mice (75), suggesting that individual HDACs have discrete, specific impact on MDSCs. Remarkably, combination therapies of inhibitors of either DNMTs or HDACs and checkpoint inhibitors (anti-PD-1 or anti-CTLA-4 antibodies) allow the eradication of checkpoint inhibitor resistant metastatic cancers by suppression of MDSCs (76). Finally, miRNAs both positively and negatively regulate the accumulation and functions of MDSCs (for instance miR-9, 17-5p, 21, 34a, 155, 181b, 210, 494, 690 vs. miR-9, 146a, 147a, 185-5p, 223, 185, 424) (70, 77). These observations, obtained in cancer models, are particularly interesting because cancer and sepsis share certain epigenetic features. Therefore, it is no surprise that oncolytic epigenetic drugs have a strong impact on innate immune responses and sepsis development (78–81). Numerous epigenetic drugs are tested in oncologic clinical trials while some are already approved for clinical applications. Altogether, these observations open a fascinating area to test epigenetic drugs targeting the expansion and/or function of MDSCs during sepsis.

## Immunosuppressive Functions of MDSCs

MDSCs suppress the activity of immune cells through various mechanisms involving the degradation of L-arginine, the production of reactive oxygen and reactive nitrogen species (ROS, RNS), the secretion of anti-inflammatory/immunosuppressive cytokines like IL-10 and transforming growth factor (TGF)-β and the activation of T regulatory cells (Tregs) (Figure 1).

L-arginine becomes a semi-essential amino acid during sepsis because of increased usage and reduced production. L-arginine shortage is sustained by the production by MDSCs of arginase that metabolizes L-arginine into L-ornithine and urea (82). L-arginine depletion affects the function of T cells through a decreased expression of the CD3 zeta-chain, which is essential for T-cell receptor (TCR) signaling (50, 83). A lack of arginase also limits the activity of natural killer (NK) cells (84). ROS, RNS, IL-10, and TGF-β skew the polarization of monocytes/macrophages and T cells toward anti-inflammatory/pro-resolving M2, Th2 and regulatory phenotypes (45, 65, 85) and impair TCR and IL-2 receptor signaling, NK cell activity and DC maturation and antigen presentation (86–89) (Figure 1). MDSCs suppress Th1 responses though direct cell-to-cell contact, but how precisely this occurs remains to be determined (45, 85). Together with CCL5/RANTES and CCL4/MIP-1β, RNS, IL-10, and TGF-β promote the recruitment and the immunosuppressive activity of Tregs, at least in cancer and in neonates (45, 85, 90, 91). The interaction between MDSCs and Tregs in sepsis is unknown.

Splenic MDSCs harvested from CLP mice early (3–5 days) and late (10 days) after sepsis onset inhibit T cell proliferation. Early MDSCs secrete less S100A9 than late MDSCs (61) and, in response to LPS and IL-6, less TNF, IL-6, IL-10, ROS, and arginase I (65). However, in response to GM-CSF, early MDSCs produce RNS and proinflammatory cytokines while late MDSCs produce arginase, IL-10 and TGF-β (69). Of note, MDSCs can also help fight infections. Indeed, MDSCs efficiently phagocytose *E. coli* and group B streptococci (92) and clear bacteria during late sepsis through a robust production of ROS (65). Thus, MDSCs have diverse biological outputs according to their surrounding milieu and sepsis progression (54, 65). More work is required to fully understand to which extend these biological variations reflect the accumulation or the differentiation of different MDSCs subpopulations during sepsis.

## Diagnostic and Prognostic Values of Immature Granulocytes and MDSCs in Human Sepsis

MDSCs make up an important proportion of immature myeloid cells. Thus, we will discuss reports analyzing immature granulocytes (IG) in adult sepsis and then move forward to studies that used more elaborated immuno-phenotyping strategies to identify MDSCs. Table 1 provides details about the design and the main observations of these studies.

**Table 1:** Studies investigating immature granulocytes and MDSCs in adults with sepsis.

**Subjects**	**Cells/phenotypes**	**Observations**	**References**
142 ED patients, 29 uninfected outpatients.	IG (automate-based determination)	Higher % in infected patients, predictor of sepsis.	(93)
70 consecutive ICU patients (51 infected, 19 uninfected).	IG (automate-based determination)	Higher % in infected patients, unrelated to day-21 and in-hospital mortality.	(94)
184 sepsis patients.	IG (automate-based determination)	Increase % associated with severity, but not predictive of mortality.	(95)
136 consecutive ICU patients.	IG (morphology and staining)	Higher % in sepsis than in uninfected patients. Unrelated to mortality.	(96)
35 sepsis and 22 non-septic consecutive burn patients, 19 healthy controls.	IG (flow cytometry)	Increase % post-burn, associated with reduced neutrophil function. Remaining elevated levels (day 7–28) associated with sepsis development	(97)
781 sepsis patients, 20 control outpatients.	IG (flow cytometry)	High % at admission related to organ failure and day-7 and day-28 mortality.	(98)
47 uninfected and 17 infected cardiac surgery patients.	IG (flow cytometry)	Increase % postoperative. Highest levels associated with secondary infection complications.	(99)
Meta-analysis (11 studies) of 1'822 sepsis patients.	Delta neutrophil index (DNI, automate-based determination)	Elevated DNI associated with mortality.	(100)
24 sepsis ICU patients, 12 hospital controls.	Interphase neutrophils (flow cytometry)	Present only in sepsis patients, proportional to sepsis severity. Suppress T-cell activity *in vitro*.	(50)
177 sepsis patients.	IG (flow cytometry)	Increase % at 48 h predictive of clinical deterioration. High % of CD10^dim^ and CD16^dim^ IG correlates with mortality. Kill activated T cells *in vitro*.	(101)
43 septic shock patients, 23 healthy controls.	IG (flow cytometry)	Increased % of CD10^dim^ and CD16^dim^ IG at days 3–4 and 6–8. Patients with lower % have better survival.	(102)
14 sepsis and 8 uninfected critically ill patients, 15 healthy controls.	M-MDSCs: SSC^low^ CD14^+^ CD11b^+^ CD16^−^ CD15^+^ PMN-MDSCs: SSC^high^ CD16^+^ CD15^+^ CD33^+^ CD66b^high^ CD114^+^ CD11b^+/low^	M-MDSCs but not PMN-MDSCs increase at day 13-21 post-sepsis. Similar % of M-MDSCs and PMN-MDSCs in sepsis and non-septic critical ill patients.	(103)
94 sepsis, 11 severity-matched ICU patients, 67 health donors.	M-MDSCs: Lin^−^ CD14^+^ HLA-DR^−/low^ PMN-MDSCs: LDG CD14^−^ CD15^+^ (Excluding eosinophils)	High % of PMN-MDSCs in sepsis patients. M-MDSCs are higher in gram-negative than gram-positive sepsis. PMN-MDSCs > 36% WBC at entry are associated with higher risk of nosocomial infections. PMN- and M-MDSCs suppress T-cell proliferation *in vitro*.	(104)
67 surgical patients with severe sepsis/septic shock, 18 healthy controls.	MDSCs: CD33^+^ CD11b^+^ HLA-DR^−^ M-MDSCs: CD14^+^ PMN-MDSCs: CD14^−^ CD15^+^	High % of MDSCs at admission correlates with early mortality. Decreasing levels of MDSCs correlate with short ICU stay. Sustained levels of MDSCs (>30% of WBC) predict nosocomial infections.	(105)
56 sepsis patients and 18 healthy controls.	M-MDSCs: CD14^+^ CD64^+^ HLA-DR^−^ PMN-MDSCs: LDG CD33^+^ CD14^neg/low^ CD64^low^ CD15^+/low^	High % of M-MDSCs in all sepsis, but particularly in gram-negative sepsis patients. Prominent PMN-MDSCs in gram-positive sepsis. PMN-MDSCs suppress T-cell proliferation *in vitro*.	(106)

*ED, emergency department; ICU, intensive care unit; IG, immature granulocytes; LDG, low density granulocytes; Lin, lineage; WBC, white blood cells*.

Accumulation of immature myeloid cells is one of the criteria established more than 25 years ago to characterize SIRS (systemic inflammatory response syndrome) and sepsis (107). The assessment of immature cells remained laborious up to the advent of automated cell counters. In an earliest study using automated IG counting on a small number of patients, the percentage of IG was higher in infected than in uninfected patients and was proposed to be a predictor of sepsis (93). Retrospective and prospective observational studies confirmed that IG proportion discriminates between infected and uninfected patients and is associated with disease severity (94–99) (Table 1). Automated cell counters can determine a delta neutrophil index (DNI), which reflects the number of immature neutrophils in the blood. A meta-analysis of ten Korean and one Egyptian studies including 1,822 sepsis patients suggests that an elevated DNI (i.e., an increased proportion of immature granulocytes) is associated with mortality (100).

Few reports demonstrate the immunosuppressive functions of immature myeloid cells in relation with sepsis and/or monitor MDSCs subpopulations using advanced flow cytometry. Since cell preparation (whole blood, with and without ficoll purification) and flow cytometry strategies are not standardized, the phenotype of MDSCs, PMN-MDSCs and M-MDSCs differs between studies (Table 1).

Gradient density interphase neutrophils arise during sepsis and their proportion correlates with disease severity in ICU patients. Cells isolated from septic shock patients deplete arginine and impair T cell functions *in vitro*, suggesting that they represent PMN-MDSCs (50). High levels of circulating CD10^dim^ CD16^dim^ IG are predictive of clinical deterioration and mortality (101, 102). This population contains a subset of CD14^−^ CD24^+^ myeloid suppressor cells that kill activated T cells *in vitro* (101).

The frequency of PMN-MDSCs (SSC^high^ CD16^+^ CD15^+^ CD33^+^ CD66b^high^ CD114^+^ CD11b^+/low^ LDG) and M-MDSCs (SSC^low^ CD14^+^ CD11b^+^ CD16^−^ CD15^+^) does not differ between non-infectious critical ill patients and sepsis patients (103). However, high levels of MDSCs are linked to nosocomial infections (Table 1). In a first study, PMN-MDSCs (CD14^−^ CD15^+^ low-density granulocytes, LDG) representing more than 36% of WBC in ICU patients sampled within 3 days of study inclusion predicts the subsequent occurrence of nosocomial infections (104). Patients that develop nosocomial infections have 2.5 times more PMN-MDSCs than patients that do not. In a second study, a close follow-up of ICU surgical patients (at days 1, 4, 7, 14, 21, and 28 or until discharge of ICU) reveals that patients with continuously high proportions of CD33^+^ CD11b^+^ HLA-DR^−/low^ MDSCs have a longer stay in the ICU, more nosocomial infections and poor functional status at discharge (105). The percentage of total MDSCs in patients with severe sepsis/septic shock raises up to 45% of WBC, and a high proportion of MDSCs at diagnosis is associated with early mortality. Comparing cell-sorted enriched CD33^+^ CD11b^+^ HLA-DR^−/low^ MDSCs from the blood of healthy subjects and septic patients reveals that pathogenic MDSCs dose dependently suppress IFNγ, IL-4, and IL-10 production by T cells more efficiently than MDSCs from healthy subjects, while healthy and disease MDSCs suppress T cell proliferation alike (105).

The proportion of PMN-MDSCs and M-MDSCs, defined as CD14^neg/low^ CD64^low^ CD15^+/low^ LDG and CD14^+^ CD64^+^ HLA-DR^neg^ leukocytes, may vary according to causative agent leading to sepsis (Table 1). M-MDSCs increase in all sepsis patients, predominantly in gram-negative cases, while PMN-MDSCs increase prominently in gram-positive sepsis (106). A subsequent study confirmed that M-MDSCs (Lin^−^ CD14^pos^ HLA-DR^low/neg^) are enriched during gram-negative sepsis, but PMN-MDSCs (CD14^−^ CD15^+^ LDG) do not differ according to the gram of the causative bacteria (104). Larger studies are required to ascertain that the microbial origin of sepsis shapes the pattern of MDSCs (108). This is an important parameter since M-MDSCs are more potent immunosuppressive than PMN-MDSCs on a per cell basis (109).

## Concluding Remarks

MDSCs play a dual role during infection and sepsis. MDSCs expanding along emergency erythropoiesis provide a first barrier against microbial invasion by producing high amounts of bactericidal molecules like ROS and RNS and counteract the hyperinflammatory response associated with early organ dysfunctions. However, MDSCs are also detrimental by supporting the establishment and/or the maintenance of a late protracted immunosuppressive environment. In line with a deletary role of MDSCs, all clinical studies to date associate high proportions of blood MDSCs with clinical worsening, occurrence of nosocomial infections and mortality of sepsis patients. Hence, MDSCs are attractive biomarkers, especially since these cells are barely detectable in healthy subjects. One limitation of clinical studies, not limited to the sepsis field, resides in the uneven phenotypic classification of MDSCs. One important future objective is to harmonize sample handling and flow cytometry strategies. Besides being attractive biomarkers, MDSCs are attractive therapeutic targets for sepsis. Inhibiting MDSCs-mediated immunosuppression or MDSCs trafficking or depleting MDSCs themselves (by normalizing myelopoiesis or inducing the differentiation of MDSCs into mature myeloid cells) would positively influence patient outcome. Interestingly, more than 30 clinical trials are running targeting MDSCs directly or indirectly in cancer patients (22). If ever envisaged for sepsis, these therapies will need specific evaluation since targeting MDSCs aggressively may put critically ill patients at risk of agranulocytosis. The results arising from these oncological studies, added to those from current or future studies in the field of sepsis, will give invaluable information onto whether and how MDSCs might be used to implement sepsis personalized medicine and precision immunotherapy.

## Author Contributions

IS and TR conceived and structured the manuscript. IS drafted the manuscript and the figure. CT revised the manuscript. TR finalized and edited the manuscript.

### Conflict of Interest Statement

The authors declare that the research was conducted in the absence of any commercial or financial relationships that could be construed as a potential conflict of interest.
